# Immunological mechanisms and antibody-drug conjugates targeting B7-H3 and B7-H4 in ovarian cancer

**DOI:** 10.3389/fimmu.2025.1683674

**Published:** 2025-11-20

**Authors:** Emily Frances Brown, Ilaria Colombo, Ainhoa Madariaga, Lawrence Kasherman

**Affiliations:** 1 Department of Medical Oncology, St George Hospital, Kogarah, NSW, Australia; 2 Department of Medical Oncology, Illawarra Cancer Care Centre, Wollongong, NSW, Australia; 3 Oncology Institute of Southern Switzerland (IOSI), Ente Ospedaliero Cantonale (EOC), Bellinzona, Switzerland; 4 Medical Oncology, Hospital Universitario 12 de Octubre, Madrid, Spain

**Keywords:** ovarian cancer, anti-body drug conjugates, B7-H3, B7-H4, tumor microenvironment, immunotherapy, drug resistance

## Abstract

Ovarian cancer remains the most lethal gynecologic malignancy, with immune evasion a major driver of therapeutic resistance and disease progression. Among novel targets, the immune checkpoint molecules B7-H3 and B7-H4 have been recognized for their potent immunosuppressive roles and selective overexpression in ovarian tumors. This review examines the immunological mechanisms shaping B7-H3 and B7-H4 activity within the ovarian tumor microenvironment, their role in facilitating immune escape, and their association with poor clinical outcomes. The development of antibody–drug conjugates targeting B7-H3 and B7-H4 offers a novel approach to deliver potent cytotoxic therapy with tumor specificity. Preclinical models and early-phase clinical studies demonstrate encouraging antitumor activity, including in treatment-resistant disease. By integrating advances in tumor immunobiology and ADC technology, this review explores how targeting B7-H3 and B7-H4 could reshape therapeutic strategies in ovarian cancer.

## 1 Introduction

Ovarian cancer remains the most lethal gynecologic malignancy, with mortality largely driven by late-stage diagnosis and acquired resistance to therapy (1, 2). Despite advances in cytoreductive surgery, chemotherapy, and targeted therapies such as poly (ADP-ribose) polymerase (PARP) inhibitors, long-term survival remains suboptimal, especially for patients with recurrent disease (3). The immunosuppressive tumor microenvironment (TME) and the ability of ovarian cancer cells to evade immune surveillance contribute significantly to disease progression and therapeutic resistance (4). Given these challenges, novel treatment strategies are urgently needed to improve patient outcomes.

Standard first-line treatment involves a combination of cytoreductive surgery and platinum-based chemotherapy (5). Maintenance therapies such as PARP inhibitors and bevacizumab, an anti-angiogenic agent, have improved progression-free survival in selected populations (6, 7). However, most patients eventually relapse and develop resistance to cytotoxic and targeted therapies, representing an urgent unmet clinical need (8, 9).

The role of the immune system in ovarian cancer is an area of increasing interest, with studies demonstrating the importance of immune checkpoint pathways, tumor-associated macrophages (TAMs) and T-cell infiltration in disease progression (10, 11). However, unlike other solid tumors such as melanoma and non-small cell lung cancer, where immune checkpoint inhibitors (ICIs) have shown substantial efficacy, ovarian cancer has displayed only modest benefit (12–14). The first large phase III trial to test ICIs in the first-line setting, IMagyn050 (atezolizumab) (15), combined an ICI with standard chemotherapy and bevacizumab but did not improve outcomes. Subsequent trials added a PARP inhibitor during maintenance to the ICI–chemotherapy backbone, including DUO-O (durvalumab) (16) and KEYLYNK 001 (pembrolizumab) (17). Both reported a progression-free survival advantage; however, interpretation is limited as the control arms did not include PARP inhibitor maintenance, now considered standard of care. More recent studies, in which PARP inhibitors were included in both arms, such as FIRST (dostarlimab) (18) and ATHENA COMBO (nivolumab) (19), have not demonstrated a meaningful clinical benefit. The reasons for the limited activity of ICIs in ovarian cancer remain unclear and are an area of ongoing investigation. Clear cell ovarian cancer may be more responsive to immune checkpoint inhibition, potentially because this subtype more frequently shows PD−L1 expression, mismatch repair deficiency, and an inflammatory tumor microenvironment. Signals of activity have been reported for pembrolizumab monotherapy (20) and for combinations such as ipilimumab plus nivolumab (21) or pembrolizumab plus lenvatinib (22). However, the only randomized trial to date in this histological subtype did not show an improvement in outcomes (23), highlighting the importance of further validation and the exploration of alternative immunotherapy strategies that can enhance tumor−specific immune responses while overcoming resistance mechanisms within the immunosuppressive tumor microenvironment.

One promising strategy in cancer therapeutics is the development of antibody–drug conjugates (ADCs). ADCs represent a new class of targeted therapeutics that combine the specificity of monoclonal antibodies with the cytotoxic potency of chemotherapy agents (24). By selectively delivering cytotoxic payloads to tumor cells expressing specific surface antigens, ADCs aim to increase intratumoral drug concentration and improve the therapeutic index (25). However, it is now clear that ADCs are not completely selective, and off−target effects and systemic toxicities remain common, reflecting the potency of the payload and the narrow therapeutic window of these agents. The therapeutic success of ADCs depends on several factors, including high and homogeneous expression of surface receptors, efficient internalization of the antibody–antigen complex, and appropriate selection of payload, linker, and drug–antibody ratio (25). Increasingly, ADCs are being investigated across a range of malignancies, including gynecologic cancers, as a novel therapeutic approach, particularly in the context of treatment resistance, where conventional chemotherapy options are limited.

Among the potential ADC targets in ovarian cancer, the immune checkpoint molecules B7-H3 (also known as CD276) and B7-H4 (also referred to as VTCN1, B7x, or B7S1) have emerged as particularly promising due to their overexpression in ovarian tumors and their role in immune evasion (26–28). B7-H3 and B7-H4 are immune regulatory proteins within the B7 family that contribute to tumor progression by inhibiting T-cell activation and fostering an immunosuppressive TME (29). Their selective overexpression in ovarian cancer, combined with their limited presence in normal tissues (30), makes them attractive candidates for ADC-based therapy.

This review examines the immunological mechanisms driving B7-H3 and B7-H4 expression in ovarian cancer, their roles in tumor immune evasion, and their potential as targets for ADCs. It discusses the current landscape of ADC development against these molecules, including preclinical and clinical progress, and addresses the challenges and future directions for integrating ADCs into ovarian cancer treatment. By highlighting the therapeutic potential of targeting B7-H3 and B7-H4, this review aims to explore how these approaches may contribute to future advances in ovarian cancer therapy.

## 2 The immunological landscape of ovarian cancer

### 2.1 The tumor-immune interface in ovarian cancer

The interaction between ovarian cancer and the immune system plays a pivotal role in disease progression and therapeutic response. A defining feature of ovarian cancer is its capacity to remodel the immune microenvironment, fostering conditions that support tumor survival and immune evasion (31). While ovarian cancer most commonly exhibits an immune “cold” or immune−desert phenotype, characterized by limited infiltration of cytotoxic immune cells (predominately CD8^+^ T cells and natural killer cells), this is not universal. A subset of tumors display an immune−excluded phenotype, and a smaller minority are immune-inflamed (“hot”), with intraepithelial T-cell infiltration. In the immune−excluded phenotype, immune cells are retained at the tumor margin and prevented from entering tumor nests by stromal barriers, abnormal vasculature, and other microenvironmental obstacles. In contrast, immune-desert tumors are largely devoid of immune infiltration (32, 33). These immune contextures influence prognosis and response to immunotherapy (34, 35). Strategies to activate pre-existing T cells may be more effective in inflamed tumors, whereas approaches to induce inflammation may be required for excluded or desert tumors.

The ovarian cancer TME is predominantly immunosuppressive (31). Regulatory T cells (Tregs) and myeloid-derived suppressor cells (MDSCs) accumulate within the ovarian TME, exerting potent immunosuppressive effects that blunt cytotoxic T cell activity and promote immune escape (11). TAMs, which comprise a significant portion of the immune infiltrate, play a critical role in shaping immune responses. In ovarian cancer, the macrophage population is skewed toward the M2 phenotype, which promotes tumor progression and immune evasion. These macrophages exist along a functional spectrum, with M1 macrophages exhibiting tumor-suppressive properties, while M2 macrophages secrete pro-tumorigenic cytokines such as VEGF, IL-10, TGF-β, and HGF (36). These cytokines enhance tumor angiogenesis and resist immune-mediated clearance while upregulating B7-H4, a potent inhibitory molecule that suppresses T-cell activation. High TAM infiltration is associated with poor prognosis (4), emphasizing their role in facilitating immune escape.

In advanced disease, malignant ascites accumulates in the peritoneal cavity, containing free-floating tumor spheroids surrounded by immune and stromal cells that contribute to immune suppression and metastasis. Within this ecosystem, immune populations, including T lymphocytes, natural killer (NK) cells and macrophages infiltrate the tumor (36). Elevated levels of tumor-infiltrating lymphocytes (TILs), especially intraepithelial CD8^+^ cytotoxic T cells, are associated with better survival, indicating a more robust anti-tumor immune response. However, ovarian cancer generally demonstrates low infiltration of these effector immune cells (33, 37). Nonetheless, certain molecular subtypes, such as BRCA−mutated and homologous recombination–deficient high−grade serous carcinomas, show greater immune cell presence and higher cytotoxic T−cell infiltration compared with homologous recombination–proficient or BRCA−wild−type tumors, which is linked to improved patient prognosis (38).

### 2.2 Mechanisms of immune evasion and checkpoint resistance in ovarian cancer

Ovarian cancer evades immune surveillance through a combination of intrinsic tumor properties and microenvironmental barriers. A low tumor mutational burden leads to fewer neoantigens, limiting T-cell recognition and contributing to poor responses to immune checkpoint blockade (39, 40). The tumor stroma, composed of fibroblasts and extracellular matrix proteins, acts as a physical barrier, excluding lymphocytes from tumor islets (4, 41). Abnormal vasculature and hypoxia further hinder immune cell trafficking and function, with hypoxia-driven upregulation of HIF-1α impairing T-cell activity (36). Malignant ascites, common in advanced disease, contains immunosuppressive cytokines such as IL-10 and TGF-β, which dampen cytotoxic responses and facilitate metastatic tumor spheroid implantation (4, 41).

Ovarian tumors upregulate inhibitory immune checkpoints, including PD-L1 (B7-H1) and CTLA-4, which suppress T-cell activation and promote exhaustion (42). Despite their success in other malignancies, immune checkpoint inhibitors (ICIs) have shown limited efficacy in ovarian cancer overall, with response rates around 10% and, in most published randomized trials to date, no improvement in survival outcomes (12, 15, 34, 43). However, emerging data from the phase III KEYNOTE B96 trial, reported in a press release, indicate an overall survival benefit with pembrolizumab combined with chemotherapy with or without bevacizumab in platinum-resistant recurrent disease, suggesting that benefit may be achievable in selected settings (44). Additional exhaustion markers such as TIM-3 and LAG-3 are frequently co-expressed on tumor-infiltrating lymphocytes (TILs), supporting the rationale for combination immune checkpoint strategies (45, 46). Tumors with high cytotoxic TIL infiltration are associated with better outcomes, while those enriched with regulatory T cells (Tregs), myeloid-derived suppressor cells (MDSCs), and M2-polarized tumor-associated macrophages (TAMs) form an immunosuppressive niche that promotes progression and treatment resistance (31, 47). Given these multifaceted mechanisms of immune escape, there is growing interest in alternative immunotherapeutic strategies, particularly those targeting novel immune checkpoints such as B7-H3 and B7-H4, which are overexpressed in ovarian tumors and contribute to immunosuppression.

## 3 B7-H3 and B7-H4: expression and function in ovarian cancer

### 3.1 Overview of the B7 family of immune checkpoint molecules

The B7 family of immune checkpoint molecules regulate adaptive immunity by modulating T-cell activation, differentiation, and tolerance. These molecules engage either costimulatory or coinhibitory receptors on immune cells, shaping the strength and direction of immune responses. While members such as B7-1 (CD80) and B7-2 (CD86) deliver activating signals through CD28, others, including B7-H3 (CD276) and B7-H4 (B7S1, VTCN1, B7x), primarily function to suppress immune activity. In cancer, aberrant expression of B7 molecules facilitates immune evasion by impairing anti-tumor T-cell responses (28, 48).

**Figure 1:**
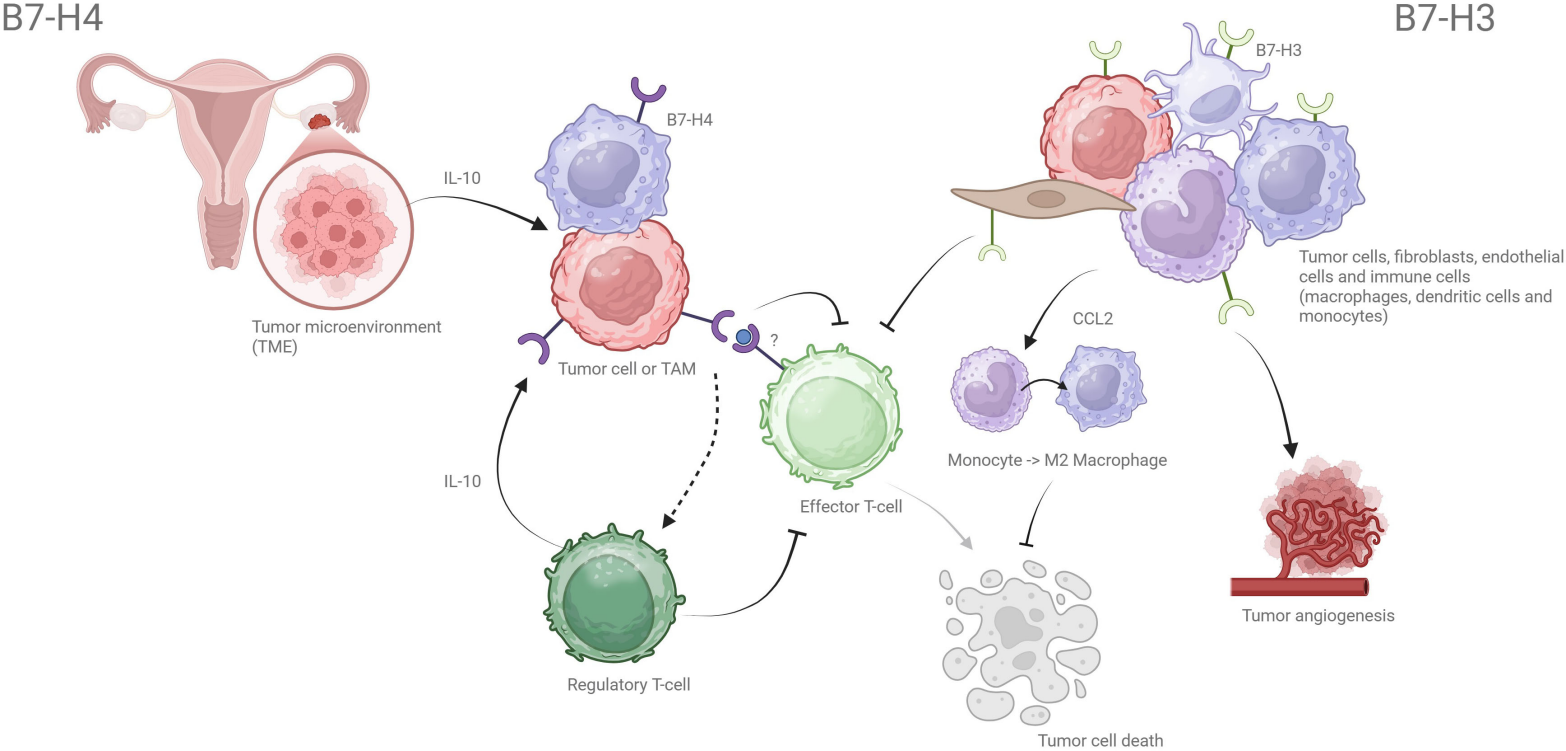
Functional roles of B7-H4 and B7-H3 in the ovarian tumor microenvironment. B7-H4 is upregulated in response to IL-10 and is primarily expressed on tumor epithelial cells and tumor-associated macrophages (TAMs). It inhibits effector T-cell activation and proliferation (through an unknown receptor), limiting their cytotoxic function and thereby impairing tumor cell killing. B7-H4 also promotes regulatory T-cell (Treg) expansion, reinforcing local immunosuppression. B7-H3, in contrast, is constitutively expressed on tumor cells, endothelial cells, and fibroblasts. Its receptor is also unknown and works to suppress both CD4^+^ and CD8^+^ T-cell activity, recruit M2-polarized TAMs via the CCL2–CCR2 axis, and support angiogenesis. **Alt Text:** Illustration showing how B7-H3 and B7-H4 contribute to immune suppression in the ovarian tumor microenvironment. B7-H4 on tumor cells and macrophages inhibit effector T cells and promote regulatory T cells. B7-H3 on tumor and stromal cells promotes M2 macrophage recruitment and tumor angiogenesis.

Of the B7 family members, B7-H3 and B7-H4 are particularly notable in ovarian cancer due to their overexpression and immunosuppressive function. Unlike classical checkpoint inhibitors such as PD-L1 and CTLA-4, which act through well-characterized receptor-ligand interactions, the precise mechanisms of action for B7-H3 and B7-H4 remain less well-defined (**Figure 1**). Notably, their receptors have not been definitively identified, yet they are known to suppress T-cell function. Their upregulation in ovarian cancer and correlation with poor prognosis suggest that they play critical roles in immune escape and tumor progression (49, 50).

### 3.2 B7-H3 and B7-H4 expression in normal tissues and physiological function

B7-H3 and B7-H4 expression is tightly regulated under normal physiological conditions, with restricted or context-dependent protein expression. Although B7-H3 mRNA is broadly detected across organs, it’s protein expression is infrequent, suggesting the existence of post-transcriptional regulatory mechanisms that limit surface expression (51). Immune cells do not constitutively express B7-H3; however, it can be induced on monocytes, dendritic cells, and T cells following stimulation with cytokines or toll-like receptor ligands (50).

Similarly, B7-H4 is largely absent from normal tissues and resting immune cells but can be upregulated on macrophages and other antigen-presenting cells (APCs) under immunosuppressive conditions, particularly in response to interleukin-10 (IL-10) (49). In contrast to other B7 family members essential for immune activation, studies in B7-H4 knockout mice indicate that B7-H4 is not required for maintaining normal immune homeostasis, supporting the idea that its function is primarily engaged in specific immunosuppressive contexts rather than routine immune regulation (52). While B7-H3 has also been implicated in non-immune processes such as bone development (35, 36), the extra tumoral functions of B7-H4 are less well characterized. Both proteins show minimal to no expression in normal ovarian tissue, as demonstrated in immunohistochemical studies (30), reinforcing their tumor-selective expression profiles and supporting their candidacy as therapeutic targets in ovarian cancer.

### 3.3 Expression patterns of B7-H3 and B7-H4 in ovarian cancer tissues

Many malignancies exploit B7 family pathways to evade immune surveillance through the overexpression of B7 ligands (53). B7-H3 and B7-H4 are highly expressed across a range of cancers, including ovarian cancer, where they are detected not only on tumor cells but also on stromal components and immune cells within the tumor microenvironment (49, 54). Their expression patterns vary across ovarian cancer histological subtypes, with studies reporting higher levels in high-grade serous carcinoma (HGSC) compared to clear cell, endometrioid, mucinous, and borderline tumors (30, 55). While inducible expression of PD-L1 (B7-H1) in response to inflammatory stimuli is well established, B7-H3 and B7-H4 demonstrate more constitutive expression, largely independent of immune activation (56). This persistent expression profile enhances their appeal as therapeutic targets.

In a landmark immunohistochemical study of 103 epithelial ovarian tumors collected between 1980 and 2004, Zang et al. systematically examined B7–H3 and B7–H4 expression (30). B7–H3 was detected in 93% of tumors and B7−H4 was detected in 100% of tumors, whereas normal ovarian tissue showed little to no staining (30). Of the 93 tumors with evaluable vasculature, 44% exhibited B7–H3 expression in the endothelium of tumor–associated blood vessels. This vascular expression was more common in serous carcinomas (62%) and in advanced–stage disease (78% of stage III/IV tumors versus 26% of stage I/II).

Several studies have linked high B7–H3 expression to poor prognosis in ovarian cancer, with increased levels correlating with advanced stage, higher tumor burden, and reduced overall survival (48, 50). Both Zang et al. and MacGregor et al. found B7–H3 on stromal fibroblasts and endothelial cells, a localization associated with poor prognosis and thought to drive tumor progression not only through immune suppression but also through pro–angiogenic effects (30, 54). Zang et al. further confirmed that endothelial B7–H3 expression correlated with advanced FIGO stage and worse disease–specific survival (30). Additionally, B7-H3 expression has been implicated in chemotherapy resistance, with *in vitro* studies showing that silencing B7-H3 sensitizes ovarian cancer cells to paclitaxel treatment (57).

B7–H4 is likewise upregulated in ovarian cancer. In the same Zang et al. cohort, this expression was broadly observed across histological subtypes without significant differences, whereas normal ovarian tissue showed negligible expression (30). B7–H4 is primarily expressed on tumor epithelial cells and TAMs, rather than on endothelial cells or other components of the tumor microenvironment (49). Unlike B7−H3, B7–H4 was not expressed in tumor vasculature in this cohort. High B7–H4 expression has been associated with poor clinical outcomes, including reduced progression–free survival and overall survival (58).

### 3.4 Functional roles of B7-H3 and B7-H4 in the ovarian tumor microenvironment

Within the ovarian TME, B7-H3 suppresses both innate and adaptive immune responses. Despite the lack of a confirmed receptor, B7-H3 consistently inhibits CD4^+^ and CD8^+^ T-cell activation, reducing cytokine production and impairing cytotoxic responses, allowing tumor cells to evade immune destruction (59, 60).

Miyamoto et al. reported that B7-H3 contributes to immune suppression by promoting the recruitment of immunosuppressive M2 macrophages via the CCL2/CCR2 axis (27), reinforcing a tumor-permissive environment. In ovarian cancer, B7-H3 antibodies have also been shown to enhance NK cell-mediated cytotoxicity in preclinical models, underscoring their potential to restore innate immune activity (61). These mechanisms highlight B7-H3’s broader role in shaping the TME and impairing anti-tumor immune responses. Beyond its immunosuppressive effects, B7-H3 is also expressed on tumor vasculature, where it contributes to angiogenesis and metastatic progression. Seaman et al. showed that targeting B7-H3 with an antibody–drug conjugate eradicated both tumor cells and the tumor vasculature, resulting in tumor regression and improved survival in preclinical models (62). Consistent with these findings, murine models lacking B7-H3 expression exhibited delayed tumor growth and prolonged survival (63).

Unlike B7-H3, which exerts pleiotropic effects, B7-H4 primarily acts as a negative regulator of T-cell activation (29). By attenuating TCR signaling and dampening costimulatory inputs, B7-H4 suppresses effector T-cell activity while promoting the expansion of Tregs, thereby shifting the immune balance toward tolerance and immune escape (64). In ovarian cancer, B7-H4 expression on TAMs is positively correlated with Treg infiltration, establishing a feedback loop that amplifies immunosuppression (64). This process is driven in part by IL-10, which induces B7-H4 expression on TAMs and further impairs cytotoxic T-cell function (64). Importantly, in preclinical models, Dangaj et al. demonstrated that B7-H4 blockade restores T-cell proliferation and enhances anti-tumor immune responses (65).

Together, B7-H3 and B7-H4 contribute to immune evasion, tumor progression, and resistance to conventional therapies. Their non-immune functions, including proliferative signaling, angiogenesis, and metabolic reprogramming, reinforce recruitment of suppressive myeloid cells, thereby sustaining an immunosuppressive tumor microenvironment. Their limited expression in normal tissues and pivotal role in shaping the immunosuppressive TME make them attractive targets for immunotherapeutic development, including antibody–drug conjugates. The key immunosuppressive functions of B7-H3 and B7-H4 in the ovarian tumor microenvironment are summarized in **Figure 1**.

## 4 Antibody–drug conjugates targeting B7-H3 and B7-H4

### 4.1 Mechanism of action of ADCs in cancer therapy

Antibody–drug conjugates (ADCs) are an emerging class of targeted cancer therapeutics that combine the specificity of monoclonal antibodies with the potent cytotoxicity of chemotherapy (24). Structurally, ADCs consist of three key components: a monoclonal antibody specific to a tumor-associated antigen, a cytotoxic payload and a chemical linker that connects the two (66). This design enables selective delivery of chemotherapy directly to tumor cells, aiming to maximize anti-tumor efficacy while minimizing systemic toxicity (25).

The monoclonal antibody component is typically a humanized or fully human IgG, selected to reduce immunogenicity and extend circulatory half-life (67). The antigen targeted by the antibody must be expressed on the surface of tumor cells, ideally with minimal expression on normal tissues to reduce off-target effects (68). Furthermore, optimal ADC efficacy depends on sufficient antigen density and efficient internalization of the antibody-antigen complex (69). Once internalized, the ADC is trafficked to the lysosome, where the linker is degraded enzymatically or chemically, releasing the cytotoxic payload intracellularly to induce cell death, usually via apoptosis (70).

Some monoclonal antibodies used in ADCs also exert intrinsic antitumor effects, either by blocking receptor-ligand interactions or by engaging immune effector mechanisms such as antibody-dependent cellular cytotoxicity, adding an immunotherapy-like dimension to their activity (71). Most therapeutic antibodies in ADCs are of the IgG1 subclass, due to their ability to mediate immune effector functions and their favorable pharmacokinetic properties (66).

The payloads used in ADCs are typically highly potent small-molecule cytotoxins, such as microtubule inhibitors (e.g., auristatins, maytansinoids) or DNA-damaging agents (e.g., duocarmycins, calicheamicins) (72). These agents are often too toxic for systemic administration without targeting (73). Many are hydrophobic and membrane-permeable, allowing for the "bystander effect," wherein the payload diffuses into adjacent tumor cells that may not express the target antigen (74). This is especially valuable in tumors with heterogeneous antigen expression. The drug-to-antibody ratio (DAR) is another critical consideration, while higher DARs may improve potency, they can compromise stability and pharmacokinetics (75, 76).

The linker plays a pivotal role in ADC performance. It must remain stable in systemic circulation to avoid premature payload release, yet be easily released in the TME or within the cell (77). Linkers can be either cleavable or non-cleavable, cleavable linkers enable bystander killing but may risk systemic toxicity if payload release occurs outside the tumor (78). Non-cleavable linkers require full internalization of the ADC followed by lysosomal degradation to release the payload, which helps minimize off-target toxicity. However, their efficacy may be reduced in tumors with heterogeneous antigen expression. Additionally, the payload-linker complex may have limited membrane permeability and be susceptible to elimination by cellular efflux pumps, potentially contributing to drug resistance (74).

ADCs represent a rapidly evolving field. As of 2025, over a dozen ADCs have received regulatory approval, with several approved in just the past four years. Until recently, no ADCs were available for gynecologic cancers. However, three ADCs are now relevant in this space: Tisotumab Vedotin was approved in 2021 for recurrent or metastatic cervical cancer (based on FDA accelerated approval) (79); Mirvetuximab Soravtansine, targeting folate receptor-alpha, was granted approval in 2024 following the MIRASOL trial, which demonstrated progression-free and overall survival benefits in patients with platinum-resistant ovarian cancer (80, 81); and Trastuzumab Deruxtecan, a HER2-directed ADC with a tumor-agnostic indication that includes HER2-positive gynecologic cancers.

### 4.2 B7-H3–directed ADCs in ovarian cancer: preclinical and clinical development

**Figure 2:**
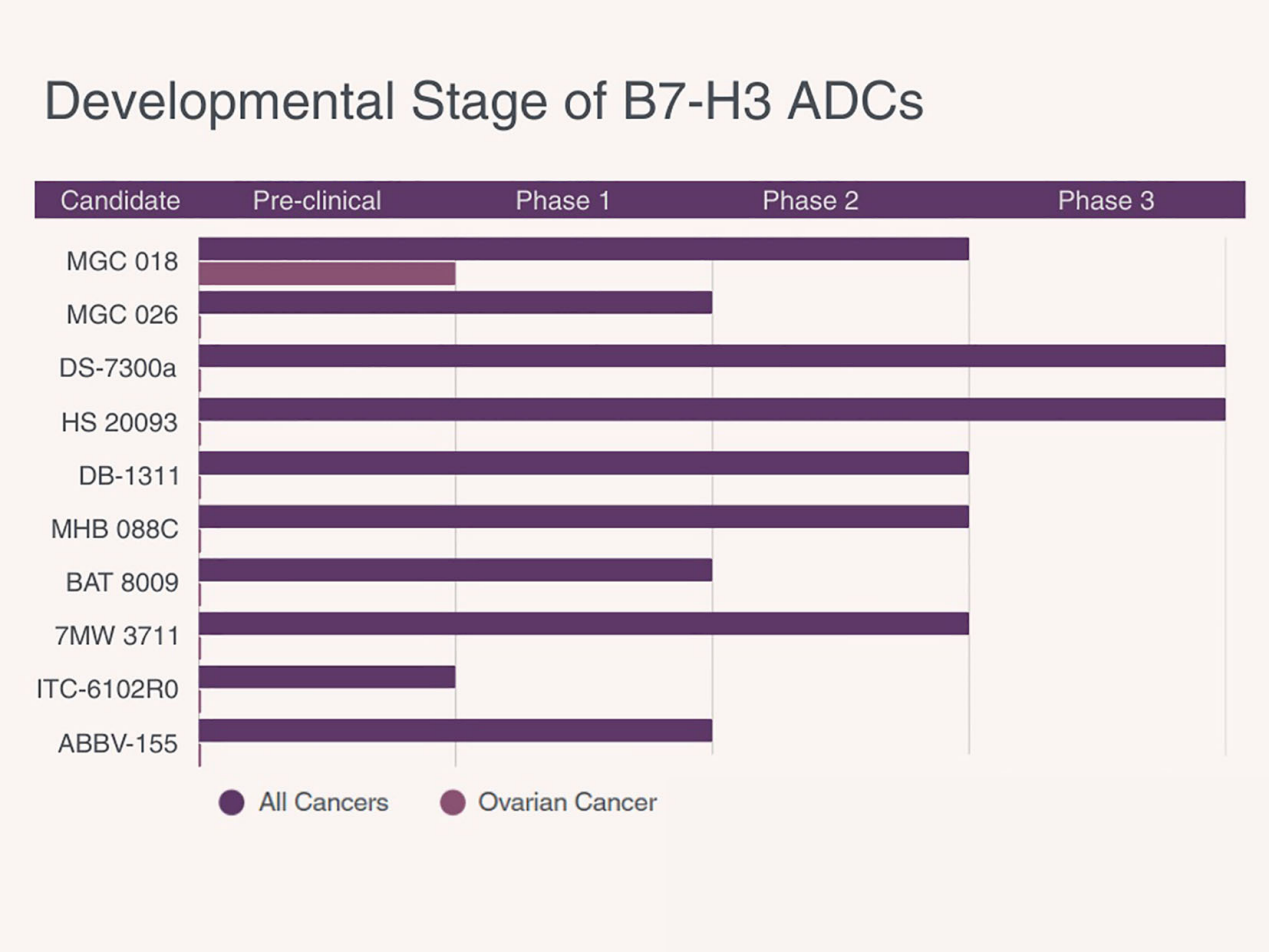
Development status of B7-H3 directed antibody–drug conjugates (ADCs) in solid tumors. Bars indicate the most advanced phase reached for each ADC as of 2025.

**Table 1** summarizes key design features of B7-H3–targeted ADCs in development, including antibody type, payload class, linker characteristics, trial phase, and evaluation in ovarian cancer. **Figure 2** depicts the current clinical development stage of each agent across cancer types.

**Table 1:** Summary of B7-H3 targeted antibody–drug conjugates (ADCs) in development, including antibody type, payload class, linker characteristics, trial phase, and evaluation in ovarian cancer. Data reflect the latest available preclinical and clinical information as of 2025. ¹Ovarian cancer may be eligible under tumor-agnostic solid tumor expansion cohorts in ongoing trials.

Agent	Antibody	Payload class	Payload name	Linker	Trial phases (years)	Tumor stream(s)	Evaluation in ovarian cancer
Vobramitamab duocarmazine (MGC018)	IgG1κ (MGA017)	DNA alkylator	Duocarmycin derivative (vc-seco-DUBA)	Cleavable – Val-Cit	Phase I (2018); Phase II (2023, mCRPC)​development discontinued	Advanced solid tumors	Preclinical efficacy shown (PA-1 xenograft model)
MGC026	IgG1κ (MGA017)	Topoisomerase I inhibitor	SYNtecan E™	Cleavable – Glycan-based	Phase I (2023 - ongoing)	Advanced/metastatic solid tumors (tumor-agnostic)	No ovarian cancer-specific data reported to date¹
Ifinatamab deruxtecan (DS-7300a)	IgG1	Topoisomerase I inhibitor	Deruxtecan (DXd)	Cleavable - Tetrapeptide	Phase I/II (2019–2023); Phase III (2024 - ongoing)	Advanced/metastatic solid tumors (tumor-agnostic); dedicated ovarian, endometrial, and cervical cohorts in PanTumor02	No ovarian cancer-specific data reported to date¹
HS-20093 (GSK'227)	IgG1	Topoisomerase I inhibitor	Undisclosed	Cleavable – Undisclosed	Phase I (2021); multiple Phase II/III trials (2023 - ongoing)	Advanced/metastatic solid tumors (tumor-agnostic); notable activity in SCLC	No ovarian cancer-specific data reported to date¹
DB-1311 (BNT324)	IgG1	Topoisomerase I inhibitor	P1021	Cleavable - Proprietary	Phase I/II (2023 - ongoing)	Advanced/metastatic solid tumors (tumor-agnostic)	No ovarian cancer-specific data reported to date¹
MHB088C	IgG1	Topoisomerase I inhibitor	SuperTopoi™	Cleavable – Undisclosed	Phase I/II (planned 2024)	Advanced/metastatic solid tumors (tumor-agnostic)	No ovarian cancer-specific data reported to date¹
BAT8009	IgG1	Topoisomerase I inhibitor	exo-Exatecan	Cleavable - Plasma-stable	Phase I (2022 - ongoing)	Advanced/metastatic solid tumors (tumor-agnostic)	No ovarian cancer-specific data reported to date¹
7MW3711	IgG1	Topoisomerase I inhibitor	Mtoxin™	Cleavable - site-specific (LysOnly™)	Phase I/II (2023 - ongoing)	Advanced/metastatic solid tumors (tumor-agnostic)	No ovarian cancer-specific data reported to date¹
ITC-6102RO	IgG1	DNA crosslinker	PBD dimer (dHBD)	Cleavable - OHPAS	Preclinical	Preclinical only	No ovarian cancer-specific data reported to date¹
Mirzotamab clezutoclax (ABBV-155)	IgG1	BCL-XL inhibitor	Undisclosed	Cleavable - solubilising	Phase I (2018 – ongoing)	Advanced/metastatic solid tumors	No ovarian cancer-specific data reported to date¹

ADC, antibody–drug conjugate; mCRPC, metastatic castration-resistant prostate cancer; SCLC, small cell lung cancer

#### 4.2.1 Vobramitamab duocarmazine (MGC018)

Vobramitamab duocarmazine (MGC018) is a B7-H3–targeting antibody–drug conjugate (ADC) comprising a humanized IgG1κ monoclonal antibody (MGA017) conjugated to vc-seco-DUBA, a duocarmycin-derived DNA-alkylating agent. The payload is linked via a protease-cleavable valine–citrulline linker with an average drug-to-antibody ratio (DAR) of ~2.7 (82). Upon binding B7-H3 and internalization, intracellular cleavage of the linker releases a membrane-permeable cytotoxic metabolite, enabling direct tumor cell killing and bystander effect.

In preclinical studies, vobramitamab duocarmazine demonstrated cytotoxicity in B7-H3–expressing tumor cell lines and potent antitumor activity in multiple xenograft models. In a PA-1 ovarian carcinoma xenograft, 10 mg/kg dosing produced 89% tumor volume reduction, including three complete regressions; lower dosing (3 mg/kg) also resulted in partial responses. Bystander killing was confirmed in mixed B7-H3+/− co-cultures. Toxicology in non-human primates indicated manageable hematological toxicity without unexpected off-target effects.

Vobramitamab duocarmazine entered a Phase I trial (NCT03729596) in 2019. Dose escalation identified 3 mg/kg Q3W as the maximum tolerated dose (83), with dose-limiting toxicities of neutropenia and fatigue. Early signs of clinical activity were reported in melanoma, triple-negative breast cancer, NSCLC, and mCRPC (83). A Phase II trial (TAMARACK) in mCRPC showed a progression-free survival of ~9.5–10 months but failed to outperform standard therapy (84), leading to termination of development in that indication. Although vobramitamab duocarmazine has not been evaluated clinically in ovarian cancer, its strong preclinical efficacy in ovarian models and mechanism of bystander killing support future exploration in this disease (85, 86).

#### 4.2.2 MGC026

MGC026 is a second-generation B7-H3–targeting antibody–drug conjugate (ADC), designed to improve upon the limitations observed with its predecessor, vobramitamab duocarmazine (87). composed of a humanized IgG1κ monoclonal antibody directed against B7-H3, conjugated to SYNtecan E, a membrane-permeable topoisomerase I inhibitor derived from the camptothecin class. The antibody is the same as that used in vobramitamab duocarmazine, but MGC026 incorporates site-specific conjugation to enable a defined drug-to-antibody ratio and reduce Fc receptor binding. This design is intended to minimize off-target uptake by immune cells such as alveolar macrophages (88).

The cytotoxic payload exerts its antitumor effect by inducing DNA damage following internalization and lysosomal cleavage. B7-H3 toxicology studies in non-human primates demonstrated a favorable safety profile with reversible toxicities observed at clinically relevant dose levels (88). These findings supported advancement to clinical evaluation, although full efficacy data from preclinical models have not been publicly disclosed.

A first-in-human Phase I trial (NCT06242470) began in late 2023 in patients with advanced solid tumors. The study is evaluating safety, tolerability, and determining a recommended dose for further development. As of early 2025, dose escalation is ongoing, and expansion into selected tumor types is expected later in the year.

#### 4.2.3 Ifinatamab deruxtecan (DS-7300a)

Ifinatamab deruxtecan (DS-7300a) is composed of a human IgG1 monoclonal antibody conjugated to a topoisomerase I inhibitor payload, deruxtecan (DXd), via a cleavable tetrapeptide-based linker (89). The DXd payload is an exatecan derivative designed to inhibit topoisomerase I, causing DNA damage and cell death in proliferating cells. Its membrane permeability enables a bystander effect, allowing cytotoxic activity in adjacent tumor cells that may have low or heterogeneous antigen expression (90).

In preclinical models, DS-7300a induced tumor regression in B7-H3–positive xenografts, with efficacy correlating to antigen density. However, because the antibody does not recognize murine B7-H3, potential effects on tumor-associated stroma or vasculature could not be assessed (91). Toxicology studies in non-human primates reported manageable safety (91).

A first-in-human Phase I/II trial, IDEATE-PanTumor01 (NCT04145622) began in 2019, enrolling patients with advanced solid tumors, including prostate, lung, and esophageal cancers. Dose escalation from 0.8 to 16 mg/kg every three weeks identified 12 mg/kg as the recommended dose (92). Adverse events included nausea, vomiting, anemia, and infusion-related reactions; one fatal case of interstitial lung disease was reported at the highest dose. Early activity was encouraging, with a disease control rate exceeding 70% (93). A subsequent trial, IDEATE-PanTumor02 (NCT06330064), is ongoing and includes dedicated cohorts for ovarian, endometrial, and cervical cancers.

#### 4.2.4 HS-20093 (GSK’227, GSK5764227)

HS-20093 (also known as GSK’227) comprises a fully human monoclonal antibody conjugated via a cleavable linker to an undisclosed topoisomerase I inhibitor payload (94). The precise structure of the linker and payload has not been publicly disclosed.

In a Phase I trial conducted in China (NCT05276609), HS-20093 was evaluated in patients with advanced solid tumors, including small-cell lung cancer (SCLC) and sarcomas. Among evaluable patients, the objective response rate (ORR) was 35%, with notable activity in SCLC: 7 of 9 patients achieved a partial response (78% ORR), including cases previously treated with camptothecin-based chemotherapy (95). The safety profile was consistent with topoisomerase I ADCs, with no new safety signals reported.

In 2022, GlaxoSmithKline licensed development rights outside of China, and a global Phase I trial (NCT06551142) commenced in 2024. This study plans to enroll approximately 280 patients with advanced solid tumors, including expansion cohorts in gastrointestinal and other malignancies. The agent has received Breakthrough Therapy designation for both SCLC and osteosarcoma (96, 97).

#### 4.2.5 DB-1311 (BNT 324)

DB-1311 (also known as BNT324) comprises a humanized IgG1 monoclonal antibody with high binding affinity and internalization capacity, conjugated via a cleavable linker to a proprietary topoisomerase I inhibitor payload (DB-1303). The payload is a camptothecin derivative designed to induce DNA damage upon intracellular release and may exert a bystander effect due to membrane permeability (98).

A multinational Phase I/II trial (NCT05914116) began in 2023 and is ongoing. As of mid-2024, 126 patients with advanced solid tumors had been treated across dose levels ranging from 3 to 12 mg/kg every three weeks. The safety profile was favorable, with low rates of severe haematological toxicities and no reported cases of interstitial lung disease. Interim efficacy data showed objective responses in multiple tumor types, with the highest activity observed in small-cell lung cancer (ORR 45.5%). Responses were also seen in metastatic castration-resistant prostate cancer (mCRPC), non-small cell lung cancer (NSCLC), and biliary tract cancers. Based on these findings, DB-1311 received FDA Fast Track designation in 2023 for unresectable or metastatic mCRPC (99).

#### 4.2.6 MHB088C

MHB088C comprises a humanized monoclonal antibody conjugated to a proprietary topoisomerase I inhibitor payload via a cleavable linker. The payload is reported to be 5–10 times more potent than conventional camptothecins (100).

A Phase I/II study conducted in China enrolled 60 patients with advanced solid tumors. Data presented at ASCO 2024 identified the maximum tolerated dose as 3.0 mg/kg administered every three weeks. The most common adverse events were mild hematological toxicities, and no cases of interstitial lung disease or severe off-target toxicity were reported. The safety profile was favorable across dose levels, including higher exposures. Preliminary efficacy signals were encouraging. Among 12 evaluable patients, the objective response rate (ORR) was 33.7%, and the disease control rate (DCR) was 91.7%. Notably, in the subgroup with small-cell lung cancer, the ORR was 100% (101).

#### 4.2.7 BAT 8009

BAT8009 is composed of a humanized monoclonal antibody conjugated to a topoisomerase I inhibitor payload via a cleavable, plasma-stable linker (102). The payload is an exatecan analogue, designed to enable membrane permeability and a bystander effect by diffusing into neighboring tumor cells following intracellular release (103). Specific details regarding the antibody format have not been disclosed.

In preclinical studies, BAT8009 demonstrated strong antitumor activity *in vitro* and in xenograft models, with a favorable safety profile (103). A Phase I dose-escalation trial (NCT05405621) commenced in August 2022 in China, enrolling patients with advanced solid tumors irrespective of B7-H3 expression. As of early 2025, the trial remains ongoing, and no efficacy results have been formally reported.

#### 4.2.8 7MW3711

7MW3711 comprises a human IgG1 monoclonal antibody conjugated to a novel topoisomerase I inhibitor (104). In preclinical studies, 7MW3711 demonstrated strong cytotoxic activity in B7-H3–expressing xenograft models and was well tolerated in non-human primates (105). Comparative studies suggested greater tumor control than earlier B7-H3 ADCs, although full data have not been publicly released. A Phase I/II clinical trial was initiated in China in 2023 (NCT06008366) to assess safety and tolerability in patients with advanced solid tumors. As of early 2025, the study remains in the dose-escalation phase, and no clinical outcomes have been reported.

#### 4.2.9 ITC-6102RO

ITC-6102RO is a B7-H3–targeted antibody–drug conjugate (ADC) comprising a humanized monoclonal antibody (SA2107) conjugated to a pyrrolobenzodiazepine (PBD) dimer, a DNA cross-linking cytotoxic agent. The linker is a cleavable ortho hydroxy-protected aryl sulfate (OHPAS), designed for high plasma stability with release triggered in the lysosomal environment. Site-specific conjugation is employed via engineered cysteine residues, yielding a homogeneous drug-to-antibody ratio of approximately 2 (106).

In preclinical studies, ITC-6102RO demonstrated selective binding and internalization into B7-H3–expressing cell lines, followed by induction of S-phase arrest, DNA damage and apoptosis. *In vivo*, it achieved potent antitumor activity in both cell-derived and patient-derived xenograft models of lung and breast cancer. At doses as low as 0.3 mg/kg, tumor growth inhibition and, in some cases, complete regressions were observed without overt toxicity (106). ITC-6102RO remains in preclinical development, with no registered clinical trials to date.

#### 4.2.10 Mirzotamab clezutoclax (ABBV-155)

Mirzotamab clezutoclax (also known as ABBV-155) comprises a fully human IgG1 monoclonal antibody conjugated via a cleavable solubilizing linker to a BCL-XL inhibitor payload (107). Unlike traditional cytotoxic payloads that disrupt microtubules or DNA, this ADC targets an anti-apoptotic protein, offering a novel mechanism of action (108). In preclinical studies, ABBV-155 demonstrated anti-tumor activity in glioblastoma models, providing rationale for clinical evaluation across B7-H3–expressing solid tumors (109). A Phase I trial (NCT03595059) initiated in 2018 is investigating mirzotamab clezutoclax in patients with relapsed or refractory solid tumors, including non-small cell lung cancer, small cell lung cancer, and breast cancer. To date, mirzotamab clezutoclax has not been evaluated specifically in ovarian cancer cohorts.

### 4.3 B7-H4–directed ADCs in ovarian cancer: preclinical and clinical development

**Figure 3:**
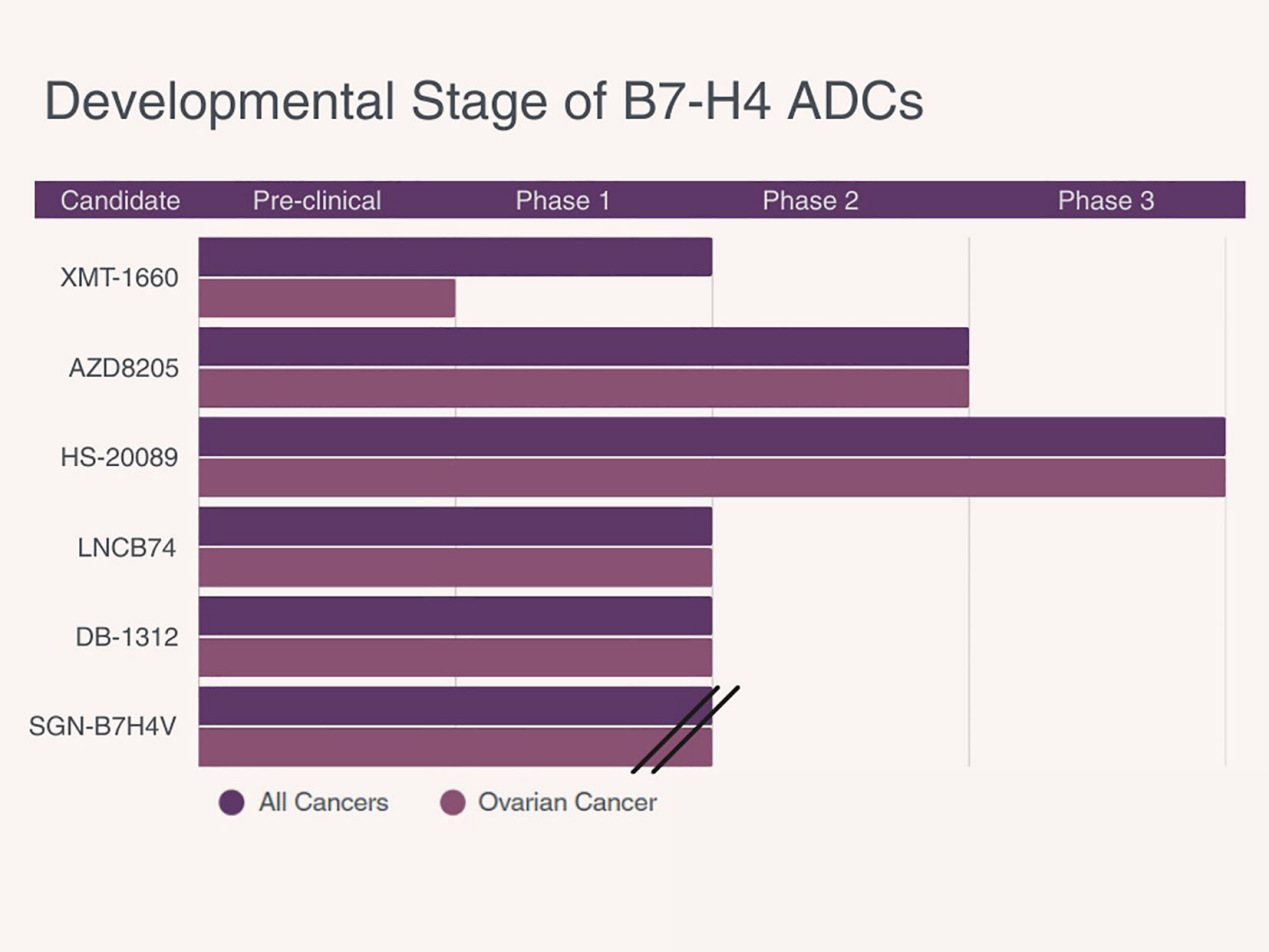
Development status of B7-H4 directed antibody–drug conjugates (ADCs) in solid tumors. Bars indicate the most advanced phase reached for each ADC as of 2025.

**Table 2** summarizes key design features of B7-H4–targeted ADCs in development, including antibody type, payload class, linker characteristics, trial phase, and evaluation in ovarian cancer. **Figure 3** depicts the current clinical development stage of each agent across cancer types.

**Table 2:** Summary of B7-H4 targeted antibody–drug conjugates (ADCs) in development, including antibody type, payload class, linker characteristics, trial phase, and evaluation in ovarian cancer. Data reflect the latest available preclinical and clinical information as of 2025.

Agent	Antibody	Payload class	Payload name	Linker	Trial phases (years)	Tumor stream(s)	Evaluation in ovarian cancer
XMT-1660	IgG1	Microtubule inhibitor	Ledadotin	Cleavable - Undisclosed	Phase I (2022)	Advanced/metastatic solid tumors (tumor-agnostic)	Preclinical efficacy shown
AZD8205	IgG1	Topoisomerase I inhibitor	AZ14170133	Cleavable - Protease (Val-Ala)	Phase I/IIa (2021-ongoing)	Advanced/metastatic solid tumors (tumor-agnostic); Phase III trial in endometrial cancer	Included in clinical trial cohorts
HS-20089	IgG1	Topoisomerase I inhibitor	Undisclosed	Cleavable - Protease	Phase I/II (2022–ongoing) Phase III planned (2025)	Advanced/metastatic solid tumors (tumor-agnostic)	Yes (Phase III planned for platinum-resistant ovarian cancer)
LNCB74	IgG1	Microtubule inhibitor	Monomethyl auristatin E (MMAE)	Cleavable	Phase I (2025 - ongoing)	Advanced/metastatic solid tumors (tumor-agnostic)	Preclinical efficacy shown, included in Phase I cohorts
BG-C9074 (DB-1312)	IgG1	Topoisomerase I inhibitor	Undisclosed	Cleavable	Phase I (2024 - ongoing)	Advanced/metastatic solid tumors (tumor-agnostic)	Included in Phase I cohorts
SGN-B7H4V	IgG1	Microtubule inhibitor	Monomethyl auristatin E (MMAE)	Cleavable (Val-Cit)	Phase I (2022) development discontinued	Advanced/metastatic solid tumors (tumor-agnostic)	Included in clinical trial cohorts

#### 4.3.1 Emiltatug ledadotin (XMT-1660)

Emiltatug ledadotin (previously XMT-1660) is a B7-H4–targeting antibody–drug conjugate (ADC) comprising a humanized IgG1 monoclonal antibody conjugated to auristatin F-hydroxypropylamide (AF-HPA), a microtubule inhibitor (110). The payload is attached via site-specific conjugation, with each antibody carrying six drug molecules in a homogeneous format (110). Upon binding to B7-H4 and internalization, AF-HPA is released intracellularly and exerts cytotoxicity, including a bystander effect due to membrane permeability. The metabolized form of AF-HPA becomes membrane-impermeable, limiting systemic toxicity (111). The antibody alone does not affect T cell proliferation, indicating that antitumor activity is driven primarily by the payload (112).

In preclinical studies, emiltatug ledadotin induced complete tumor regressions in xenograft models of breast and ovarian cancer, including immune-refractory settings (110). A Phase I trial (NCT05377996) began in 2022 in patients with advanced solid tumors, including triple-negative breast, endometrial, and ovarian cancers. Interim data showed confirmed responses in 23% of patients with high B7-H4 expression (113). The agent has received FDA Fast Track designation for HER2-low/negative breast cancer (114).

#### 4.3.2 AZD8205 (Puxitatug samrotecan)

AZD8205 (puxitatug samrotecan) is a B7-H4–targeted antibody–drug conjugate composed of a humanized IgG1 monoclonal antibody conjugated to AZ14170133, a proprietary camptothecin-derived topoisomerase I inhibitor. The payload is attached via a valine–alanine dipeptide linker with a PEG8 spacer, designed for lysosomal cleavage and intracellular release. Once liberated, the payload induces DNA damage through topoisomerase I inhibition and is membrane-permeable, enabling a bystander effect in neighboring tumor cells (115).

In preclinical studies, AZD8205 induced tumor regressions in 69% of B7-H4–positive patient-derived breast cancer xenografts, including models with heterogeneous antigen expression. It also demonstrated synergistic antitumor activity in BRCA-mutated models when combined with a PARP inhibitor (115). A Phase I/II trial (NCT05123482), initiated in 2021, is ongoing in patients with advanced solid tumors. Interim results reported partial responses in 9 of 47 heavily pretreated patients, including individuals with platinum-resistant ovarian cancer, with a manageable safety profile primarily involving hematological toxicities (116). Expansion cohorts are underway in ovarian, breast, endometrial, and biliary tract cancers.

#### 4.3.3 HS-20089 (GSK5733584)

GSK5733584 (also known as HS-20089) is a B7-H4–targeted antibody–drug conjugate (ADC) incorporating a humanized IgG1 monoclonal antibody conjugated via a protease-cleavable linker to a topoisomerase I inhibitor payload (117). The precise structure of the linker-payload has not been publicly disclosed. GSK obtained rights to the compound outside of Asia through a 2023 partnership with Hansoh (118).

In a global phase I dose-escalation study (NCT05263479), HS-20089 demonstrated encouraging clinical activity in advanced solid tumors. Among 16 patients with triple-negative breast cancer (TNBC), the objective response rate (ORR) was 37.5%, with a higher ORR of 41.7% observed in those treated at presumed target doses of 4.8 and 5.8 mg/kg every three weeks. Two patients with ovarian cancer were enrolled, although specific response data for this subgroup were not reported. The safety profile was consistent with other topoisomerase I inhibitor–based ADCs, with common adverse events including neutropenia and gastrointestinal symptoms, which were generally manageable (119).

GSK5733584 is currently undergoing global Phase I evaluation (NCT06431594), while a separate Hansoh-sponsored program in China (NCT06855069) has progressed to a randomized Phase III trial in platinum-resistant epithelial ovarian, fallopian tube, and primary peritoneal cancers. The trial randomizes patients to HS-20089 or investigator’s choice of single-agent chemotherapy (paclitaxel, doxorubicin, or topotecan). Key eligibility criteria include platinum-resistant disease, measurable lesions per RECIST 1.1 and an ECOG performance status of 0–1. Notably, B7-H4 expression testing is not required for enrollment. If successful, it could lead to the first approval of a B7-H4–directed ADC, specifically in ovarian cancer (120). The decision to pursue Phase III development in this setting highlights the strength of the early clinical signal and the potential relevance of B7-H4 as a therapeutic target in platinum-resistant disease.

#### 4.3.4 LNCB74

LNCB74 is a B7-H4–targeting antibody–drug conjugate (ADC) consisting of a human IgG monoclonal antibody linked to the cytotoxic agent monomethyl auristatin E (MMAE) via a β-glucuronide cleavable linker. The design enables selective release of the drug in the TME, where the enzyme β-glucuronidase is present, while maintaining stability in circulation (121). This approach aims to improve tumor specificity and reduce toxicity to normal tissues.

In preclinical studies, LNCB74 showed strong anti-tumor activity in B7-H4–expressing models, including breast, endometrial, and ovarian cancers. It produced complete tumor regressions in several mouse models. In preclinical studies, LNCB74 demonstrated a favorable safety profile, with good tolerability observed in nonhuman primates following repeat dosing (122).

A first-in-human Phase I trial (NCT06774963) began in 2025, enrolling patients with advanced solid tumors, including ovarian, breast, and endometrial cancers. The trial includes retrospective analysis of tumor B7-H4 expression to guide future patient selection.

#### 4.3.5 DB-1312 (BG-C9074)

DB-1312 (also known as BG-C9074) is a B7-H4–targeting antibody–drug conjugate (ADC) comprising a humanized IgG monoclonal antibody conjugated to a camptothecin-derived topoisomerase I inhibitor payload via a cleavable linker (120, 123). Preclinical data specific to DB-1312 have not been published. A Phase I trial of DB-1312 (NCT06233942) alone or in combination with Tislelizumab commenced in 2024 in patients with advanced solid tumors, following BeiGene’s global licensing of the compound in mid-2023. The trial is in dose escalation and aims to determine safety, tolerability, and early efficacy, with initial readouts anticipated in 2025. Although no clinical data in ovarian cancer has been reported, DB-1312 is being tested in a tumor-agnostic Phase I trial.

#### 4.3.6 Felmetatug vedotin (SGN-B7-H4V)

Felmetatug vedotin (previously SGN-B7H4V, PF-08046048) was a vedotin-based B7-H4 ADC comprising a fully human IgG1 antibody linked to monomethyl auristatin E via a cleavable mc-vc linker (124). It entered Phase I trials (NCT05194072) for breast, ovarian, and endometrial cancers but was discontinued in early 2024 due to limited efficacy relative to chemotherapy, despite preclinical activity in B7-H4–positive ovarian cancer models.

## 5 Challenges and future directions

### 5.1 Potential resistance mechanisms to ADCs targeting B7-H3 and B7-H4

Despite their promise, antibody–drug conjugates (ADCs) targeting B7-H3 and B7-H4 may encounter multiple resistance mechanisms that limit therapeutic efficacy in ovarian cancer. These include tumor-intrinsic adaptations, microenvironmental barriers, and immune-mediated escape (125).

Antigen downregulation and heterogeneity are major contributors to resistance. Tumor cells may evade ADCs by reducing or losing expression of B7-H3 or B7-H4, or by selecting for antigen-negative subclones (126). Although B7-H4 is commonly overexpressed in HGSC and remains expressed after chemotherapy and PARP inhibitors (127, 128), ADC-induced selective pressure could drive antigen loss. Intra tumoral heterogeneity may further enable survival of low-expressing cells. Bystander-capable payloads, such as duocarmycin or membrane-permeable topoisomerase inhibitors, may help overcome this challenge by diffusing into adjacent antigen-negative cells (85).

Impaired internalization and trafficking of the ADC–antigen complex also reduces efficacy. Tumor cells may reroute endocytosis through less efficient pathways, such as caveolin-mediated uptake, or alter glycosylation patterns that trap ADCs on the cell surface (129, 130). Impaired lysosomal function, such as altered pH or reduced protease activity, can hinder the effective release of the cytotoxic payload (125). In addition, soluble B7-H4 present in the circulation may act as an antigen sink, binding ADCs and reducing their availability for tumor targeting (131).

Efflux of payloads by ATP-binding cassette (ABC) transporters such as ABCB1 (P-glycoprotein) and ABCC1 (MRP1) is another resistance mechanism, particularly in chemotherapy-pretreated ovarian cancers (125, 132). These pumps can expel cytotoxic agents, diminishing ADC efficacy. Payload selection is critical; newer agents employ efflux-resistant payloads like exatecan derivatives to retain potency in multidrug-resistant contexts (133, 134).

Tumor cells may also acquire resistance to the payload. Mutations in topoisomerase I, altered tubulin isoform expression, and enhanced DNA repair capacity through pathways such as homologous recombination or ATM/ATR signaling can reduce susceptibility (135–137). Overexpression of anti-apoptotic proteins like BCL-2 may further blunt the cytotoxic response (125).

The TME presents additional challenges. Dense stroma, high interstitial pressure, and hypovascularity can limit ADC penetration, particularly for large antibody molecules (125, 138). B7-H3 expression on stromal cells may create a binding site barrier, trapping the ADC in peripheral tumor zones (54). Acidic or protease-rich extracellular conditions may trigger premature payload release (125).

Immune-related mechanisms may influence long-term efficacy. Although B7-H3 and B7-H4 contribute to immune suppression, their removal may unmask compensatory pathways such as PD-L1 upregulation or immunosuppressive cytokine production (139). In some cases, anti-drug antibodies (ADAs) may emerge with repeated exposure, accelerating ADC clearance (140). These observations underscore that targeting B7-H3 or B7-H4 alone may be insufficient to overcome redundant immunosuppressive pathways, highlighting the importance of rational combination approaches.

### 5.2 Approaches to enhance the efficacy of B7-H3 and B7-H4 ADCs

Combination strategies are being actively explored to enhance the antitumor efficacy of B7-H3 and B7-H4 ADCs in ovarian cancer, aiming to improve tumor cell killing, overcome resistance, and promote durable immune responses.

Immune-based combinations are a major area of interest. BG-C9074 is being evaluated with tislelizumab (anti–PD-1; NCT06233942), GSK-5733584 is being studied alongside adebrelimab (anti–PD-L1) and bevacizumab (anti–VEGF; NCT06431594), and the BLUESTAR trial (NCT05123482) is exploring puxitatug samrotecan with rilvegostomig, a PD-1/TIGIT bispecific antibody. These combinations aim to enhance T cell–mediated responses and counteract immune suppression within the TME.

In parallel, preclinical studies combining B7-H3 ADCs with co-stimulatory agonists (e.g., 4-1BB) or bispecific T-cell engagers have demonstrated enhanced immune activation (141, 142). Experimental strategies such as tumor vaccines and oncolytic viruses are also being investigated to further promote antigen release and immune priming (143).

Given the DNA-damaging mechanisms of many ADC payloads and the high prevalence of homologous recombination deficiency in ovarian cancer, combining ADCs with PARP inhibitors is particularly compelling. In patient-derived xenograft models, B7-H4–directed ADCs combined with AZD5305, a selective PARP1 inhibitor, achieved complete tumor control, including in low antigen–expressing tumors (115). AZD8205 is currently undergoing clinical evaluation both as monotherapy and in combination with PARP inhibitors (NCT05123482).

Optimizing tumor delivery is another strategy under investigation. Pairing ADCs with anti-angiogenic agents such as bevacizumab may improve vascular perfusion and enhance ADC access to tumor tissue (144, 145).

While B7-H3 and B7-H4 ADCs are still in phase I/II development, early activity signals, particularly in platinum-resistant and post-PARP settings, support continued exploration. Future progress will depend on validating combinations that show superiority over non-platinum chemotherapy ± bevacizumab. Biomarker development, including companion diagnostics and longitudinal antigen profiling, will be essential for guiding patient selection and treatment adaptation.

## 6 Conclusion

The immune checkpoint molecules B7-H3 and B7-H4 have emerged as promising therapeutic targets in ovarian cancer, offering a new avenue to address the persistent challenges of immune evasion and treatment resistance. These molecules are overexpressed in most HGSC and are associated with poor prognosis, immunosuppression, and disease progression. ADCs targeting B7-H3 and B7-H4 offer a novel mechanism to selectively deliver cytotoxic agents to ovarian tumors, with the potential to improve therapeutic efficacy while minimizing off-target toxicity.

A diverse pipeline of agents utilizing duocarmycin, topoisomerase I inhibitors, PBD dimers, and other payloads, are currently under early-phase clinical investigation. Preliminary efficacy signals are encouraging, and the field is entering a pivotal phase in which upcoming trials will evaluate whether these ADCs can meaningfully improve outcomes in defined patient populations. Platinum-resistant ovarian cancer represents a key unmet need where such therapies may offer particular benefit. Exploratory studies of B7-H3 and B7-H4 ADCs, either as monotherapy or in combinations (e.g., with PD-1 inhibitors), are anticipated. However, clinical success will require careful attention to resistance mechanisms, optimal sequencing strategies, and biomarker-driven patient selection.
